# Angiotensin II-induced Hypertension is Reduced by Deficiency of P-selectin Glycoprotein Ligand-1

**DOI:** 10.1038/s41598-018-21588-3

**Published:** 2018-02-19

**Authors:** Qian Wang, Hui Wang, Jintao Wang, Jessica Venugopal, Kyle Kleiman, Chiao Guo, Yingxian Sun, Daniel T. Eitzman

**Affiliations:** 1 Department of Cardiology, the First Hospital of China Medical University, Shenyang, China; 2 Department of Internal Medicine, Cardiovascular Research Center, University of Michigan, Ann Arbor, Michigan USA

## Abstract

Identification of inflammatory mediators that regulate the vascular response to vasopressor molecules may aid in the development of novel therapeutic agents to treat or prevent hypertensive vascular diseases. Leukocytes have recently been shown to be capable of modifying blood pressure responses to vasopressor molecules. The purpose of this study was to test the hypothesis that deficiency of the leukocyte ligand, Psgl-1, would reduce the pressor response to angiotensin II (Ang II). Mice deficient in Psgl-1 (*Psgl-1*^−/−^) along with wild-type (WT) controls were treated for 2 weeks with a continuous infusion of Ang II. No differences in blood pressure between the groups were noted at baseline, however after 5 days of Ang II infusion, systolic blood pressures were higher in WT compared to *Psgl-1*^−/−^ mice. The pressor response to acute administration of high dose Ang II was also attenuated in *Psgl-1*^−/−^ compared to WT mice. Chimeric mice with hematopoietic deficiency of Psgl-1 similarly showed a reduced pressor response to Ang II. This effect was associated with reduced plasma interleukin-17 (IL-17) levels in *Psgl-1*^−/−^ mice and the reduced pressor response was restored by administration of recombinant IL-17. In conclusion, hematopoietic deficiency of Psgl-1 attenuates Ang II-induced hypertension, an effect that may be mediated by reduced IL-17.

## Introduction

Hypertension is associated with biomarkers of inflammation in humans^1^ and preclinical studies have demonstrated a causal role for both innate and adaptive immune responses towards blood pressure regulation^2^. Mice deficient in both T and B lymphocytes demonstrate an attenuated pressor response to angiotensin II (Ang II), an effect that is restored with reconstitution of T cells^3^. T cell production of interleukin-17 (IL-17) appears to be a significant mediator of this effect^4^. IL-17 has also been shown to enhance leukocyte chemotactic responses^5,6^ and to enhance cellular responses to other inflammatory cytokines^7^.

P-selectin glycoprotein ligand-1 (Psgl-1) is a leukocyte ligand that binds to selectins and mediates tissue recruitment of leukocytes and platelets^8^. Psgl-1 is required for sequential recruitment and generation of Th17 T cells in some models of inflammation^9^ suggesting Psgl-1 could contribute to regulation of vascular tone. Deficiency of Psgl-1 has previously been shown to lead to endothelial cytokine resistance due to attenuation of endothelial NF-κB activation^10^. Since Ang II has also been shown to signal through NF-κB^11^, we tested the hypothesis that deficiency of Psgl-1 would attenuate the pressor response to Ang II.

## Methods

### Animals

Male C57BL6/J and *Psgl-1*^−/−^ mice were originally purchased from the Jackson Laboratory (Bar Harbor, Maine). *Psgl-1*^−/−^ mice were backcrossed to the C57BL6/J strain >16 generations before use in these experiments. Mice were housed under specific pathogen-free conditions in static microisolator cages and fed with standard laboratory rodent diet (No. 5001, TestDiet, Richmond, IN) and tap water ad libitum in a temperature-controlled room with a 12:12-hour light/dark cycle. All animal use protocols complied with the Principles of Laboratory and Animal Care established by the National Society for Medical Research and were approved by the University of Michigan Committee on Use and Care of Animals.

### Ang II-induced hypertension model

After baseline blood pressure recordings, mice were anesthetized and underwent subcutaneous implantation of osmotic minipumps (model 2004, Alzet, Cupertino, CA). Saline or Ang II (Sigma, St. Louis, MO) was continuously delivered at an infusion rate of 500 ng/kg/min for two weeks. Blood pressures were measured in non-anesthetized mice by tail plethysmography using the BP-2000 Blood Pressure Analysis System (Visitech System, Apex, NC) every day for two weeks. To get reliable and stable pressure measurements, mice were trained for seven consecutive days before implantation of minipumps. All blood pressure measurements were consistently performed in the morning.

Blood pressure was also measured invasively via carotid arterial catheterization as previously described^12^. Briefly animals were anesthetized with urethane (1.0 g/kg, intraperitoneally) while body temperature was maintained at 37 °C on a controlled heating pad. After clearing surrounding tissue from the right common carotid artery, an arteriotomy was performed using fine scissors. A 1.4 F micro-tip catheter sensor (model SPR-671, Millar Instruments InC., Houston, TX) was inserted into the carotid artery toward the heart and blood pressure was equilibrated for 10 minutes to reach a steady state. Then cumulative doses of Ang II (0.2–200 μg/kg) were administrated via the jugular vein by a GENIE Plus Infusion Syringe Pump (Kent Scientific, Torrington, CT). Blood pressure was recorded using a data acquisition system powerlab 8/30 and chart software (AdInstruments, Colorado Springs, CO).

### Psgl-1 neutralizing antibody experiments

For Psgl-1 antibody experiments, a rat anti-mouse Psgl-1 antibody (4RA10)^13^ or isotype control rat IgG_1_ k (100 µg in 200 µl PBS) (BD Biosciences, San Jose, CA) was injected into 8-week-old WT mice intraperitoneally. 16 hours following injection, the pressor response to Ang II was determined via invasive carotid artery blood pressure monitoring.

### IL-17 treatment

For IL-17 replacement experiments, recombinant murine IL-17 (1000 ng in 200 µl PBS) (PeproTech, Rocky Hill, NJ) or PBS was injected into 8-week-old *Psgl-1*^−/−^ mice. 5 hours later, the pressor response to Ang II was determined via invasive carotid artery blood pressure monitoring. Dosing was based on a previous study^14^.

### Bone marrow transplantation (BMT)

To determine the contribution of hematopoietic Psgl-1 towards blood pressure regulation, mice chimeric for Psgl-1 were generated by BMT^15^. Briefly, WT mice were placed on acid water 2 weeks prior to BMT and then used as recipients for WT or *Psgl-1*^−/−^ donors. Bone marrow was harvested from donor mice by flushing femurs and tibias with RPMI 1640 medium containing 2% fetal bovine serum. Cells were then centrifuged at 300 *g* and resuspended in phosphate-buffered saline before injection. Recipient mice were irradiated (2 × 650 rad [0.02 × 6.5 Gy]) and then injected with 4 × 10^6^ bone marrow cells from WT or *Psgl-1*^−/−^ mice via the tail vein. 8 weeks after BMT, the mice were used for blood pressure monitoring via carotid artery catheterization.

### Enzyme-linked immunosorbent assay (ELISA)

Plasma samples were collected via ventricular puncture at the time of euthanasia for measurement of sP-sel, sE-sel, and IL-17 using commercially available murine ELISA kits (R&D Systems, Minneapolis, MN) according to manufacturers’ instructions.

### Statistical analysis

All data are presented as mean ± standard error. Statistical analysis was performed using GraphPad Prism. Results were analyzed using unpaired t test for comparison between 2 groups. For multiple comparisons, results were analyzed using 1-way ANOVA followed by Tukey post-test analysis. P values < 0.05 were considered statistically significant.

## Results

### Blood pressure response to Ang II is attenuated in *Psgl-1* deficient mice

**Figure 1:**
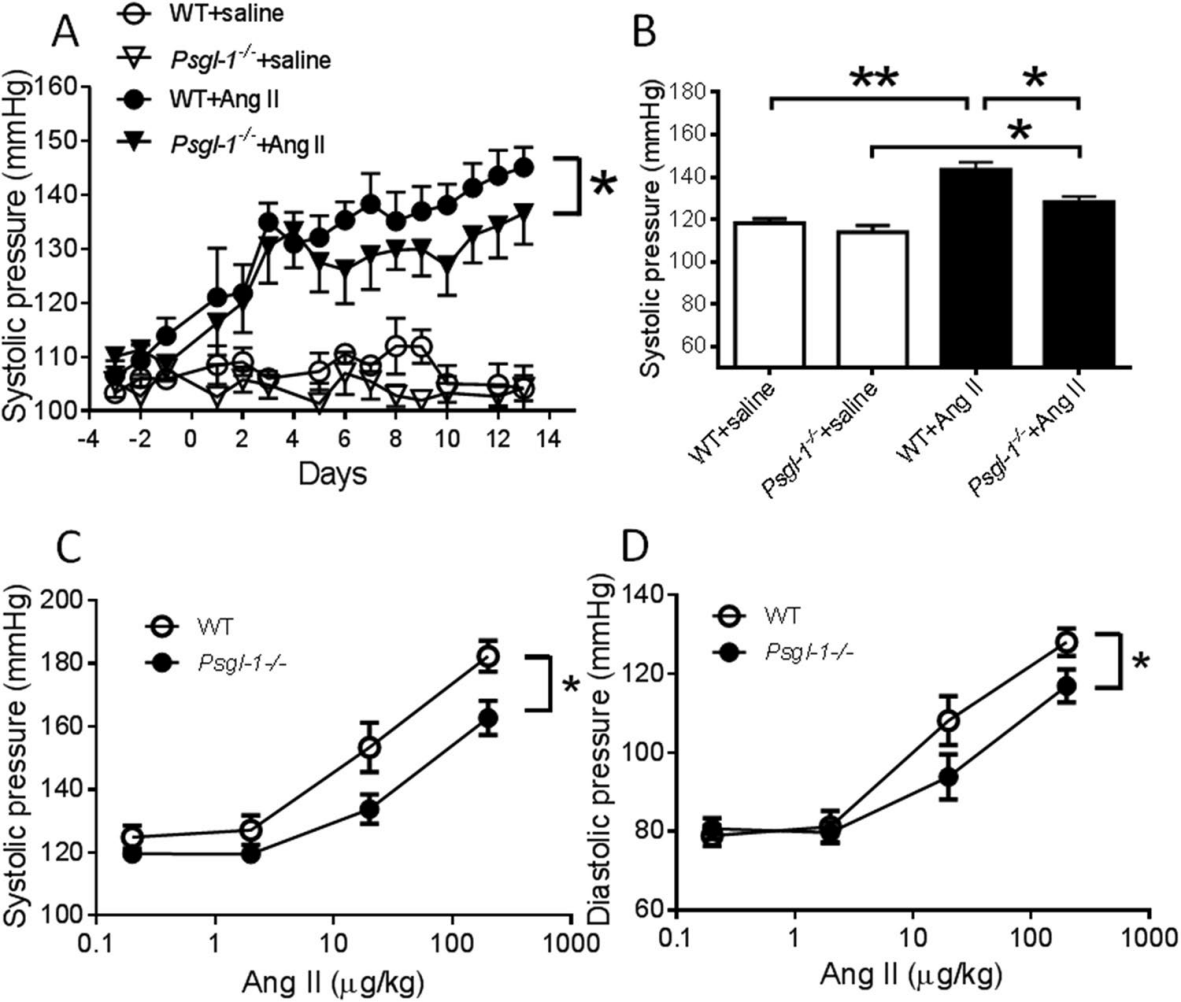
Effect of Psgl-1 deficiency on Ang II-induced hypertension. (**A**) Tail-cuff measurement of systolic blood pressure of WT and *Psgl-1*^−/−^ mice in response to chronic infusion of saline or Ang II administered via osmotic minipumps (n = 10 mice per group). (**B**) Systolic blood pressure measured following catheterization of the carotid artery in response to chronic infusion of saline or Ang II (n = 5 mice per group). (**C**) Systolic blood pressure response to acute infusion of Ang II in WT and *Psgl-1*^−/−^ mice (n = 5 mice per group). (**D**) Diastolic blood pressure response to acute infusion of Ang II in WT and *Psgl-1*^−/−^ mice. *P < 0.05.

To test the hypothesis that deficiency of Psgl-1 would reduce Ang II-induced hypertension, saline or Ang II was chronically administrated via osmotic minipumps into C57BL6/J (wild-type, WT) or *Psgl-1*^−/−^ mice for 2 weeks. At baseline and with infusion of only saline, blood pressure was similar between WT and *Psgl-1*^−/−^ mice. Systemic infusion of Ang II increased blood pressure as measured by tail-cuff plethysmography and carotid artery catheterization in both WT and *Psgl-1*^−/−^ mice compared with saline-infused control mice. However, by infusion day 4 the increase in blood pressure was significantly attenuated in *Psgl-1*^−/−^ mice compared to WT mice (Fig. 1A and B). To determine whether this was due to acute or chronic effects of Ang II on the vasculature, the acute effect of high dose Ang II on blood pressure was assessed. For these experiments the pressor response to Ang II was measured in anesthetized mice by invasive blood pressure recording using carotid artery catheterization. Cumulative Ang II (0.2, 2, 20, 200 μg/kg) was administrated via the jugular vein with a 5 minute interval between increasing doses. The systolic and diastolic blood pressures were then recorded in response to the varying doses of Ang II. In *Psgl-1*^−/−^ mice, the response to Ang II on systolic and diastolic blood pressure was significantly reduced compared to WT mice (Fig. 1C and D) indicating Psgl-1 deficiency attenuates both chronic and acute effects of Ang II.

### Circulating biomarkers are reduced in Psgl-1 deficient mice following Ang II challenge

**Figure 2:**
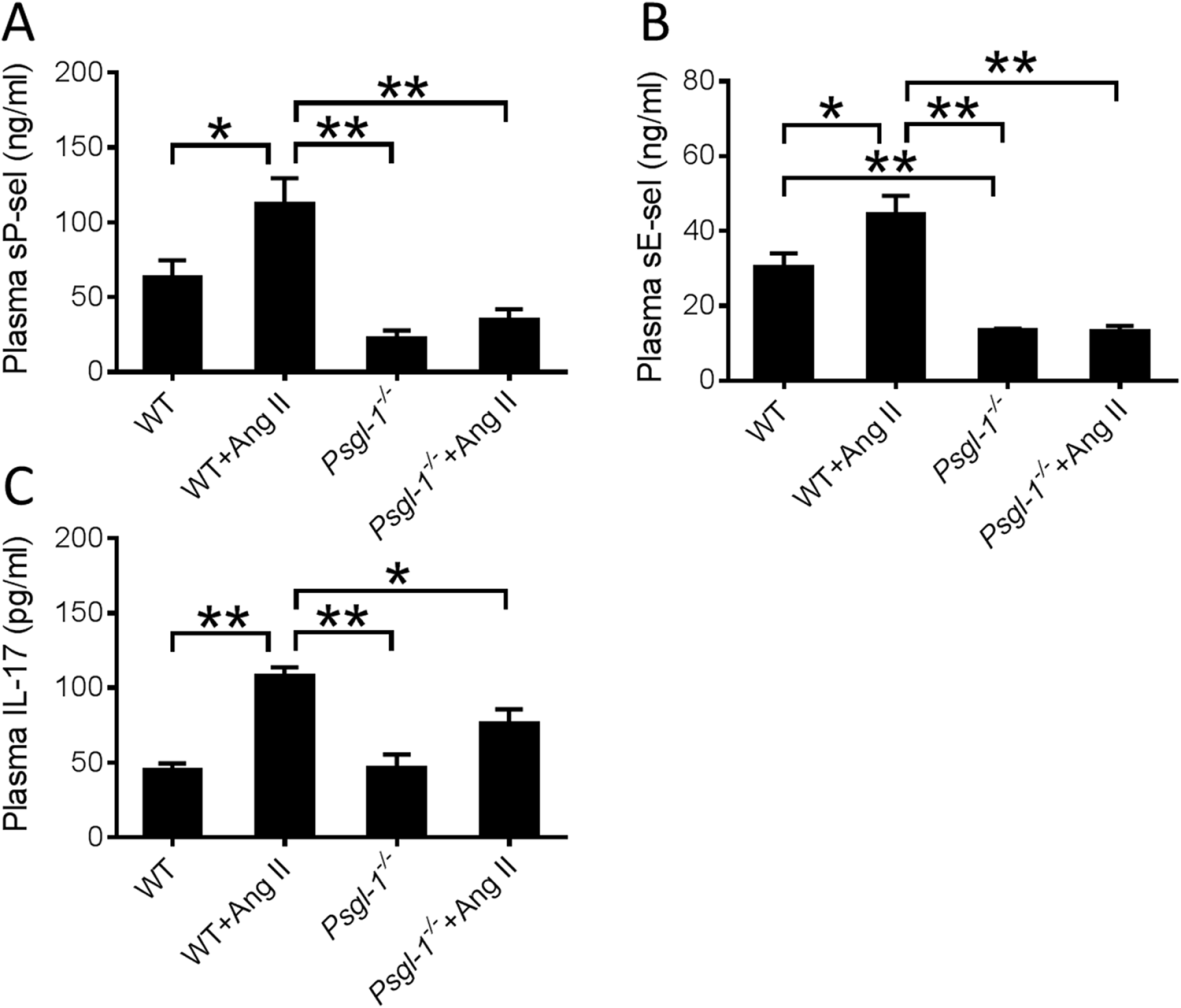
Levels of plasma factors before and after cumulative infusion of Ang II in WT and *Psgl-1*^−/−^ mice (n = 5 mice per group). (**A**) Levels of soluble P-selectin (sP-sel). (**B**) Levels of soluble E-selectin (sE-sel). (**C**) Levels of interleukin-17 (IL-17). *P < 0.05. **P < 0.01.

To determine the effect of Psgl-1 deficiency on a group of relevant circulating biomarkers, the plasma levels of soluble P-selectin (sP-sel), soluble E-selectin (sE-sel), and IL-17 were measured in WT and *Psgl-1*^−/−^ mice before and after Ang II administration. After infusion of the consecutive doses of intravenous Ang II infusions and blood pressure measurements, plasma samples were collected and assayed for sP-sel, sE-sel and IL-17. These molecules are all products of NF**κ**B target genes^16–19^. Consistent with our hypothesis, each of these proteins was lower in *Psgl-1*^−/−^ compared to WT mice (Fig. 2A,B,C), consistent with reduced NF**κ**B signaling in the setting of Psgl-1 deficiency.

### Hematopoietic Psgl-1 pool mediates effects of Psgl-1 deficiency towards blood pressure

**Figure 3:**
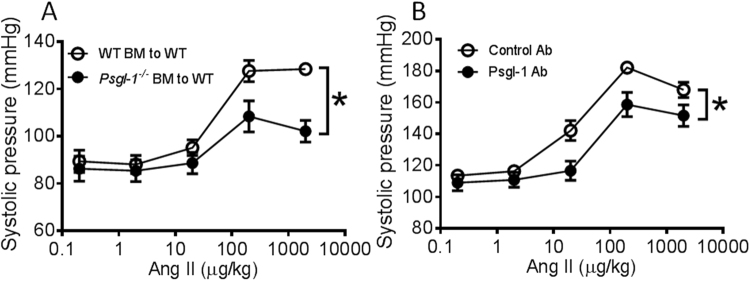
Pressor responses to acute infusion of Ang II after bone marrow transplantation or Psgl-1 antibody treatment (n = 5 mice per group). (**A**) Pooled data of systolic blood pressure response to Ang II in WT mice receiving WT bone marrow (BM) and WT mice receiving *Psgl-1*^−/−^ BM. (**B**) Pooled data of systolic blood pressure response to Ang II in WT mice receiving control antibody (Ab) and WT mice receiving anti-Psgl-1 Ab. *P < 0.05.

**Figure 4:**
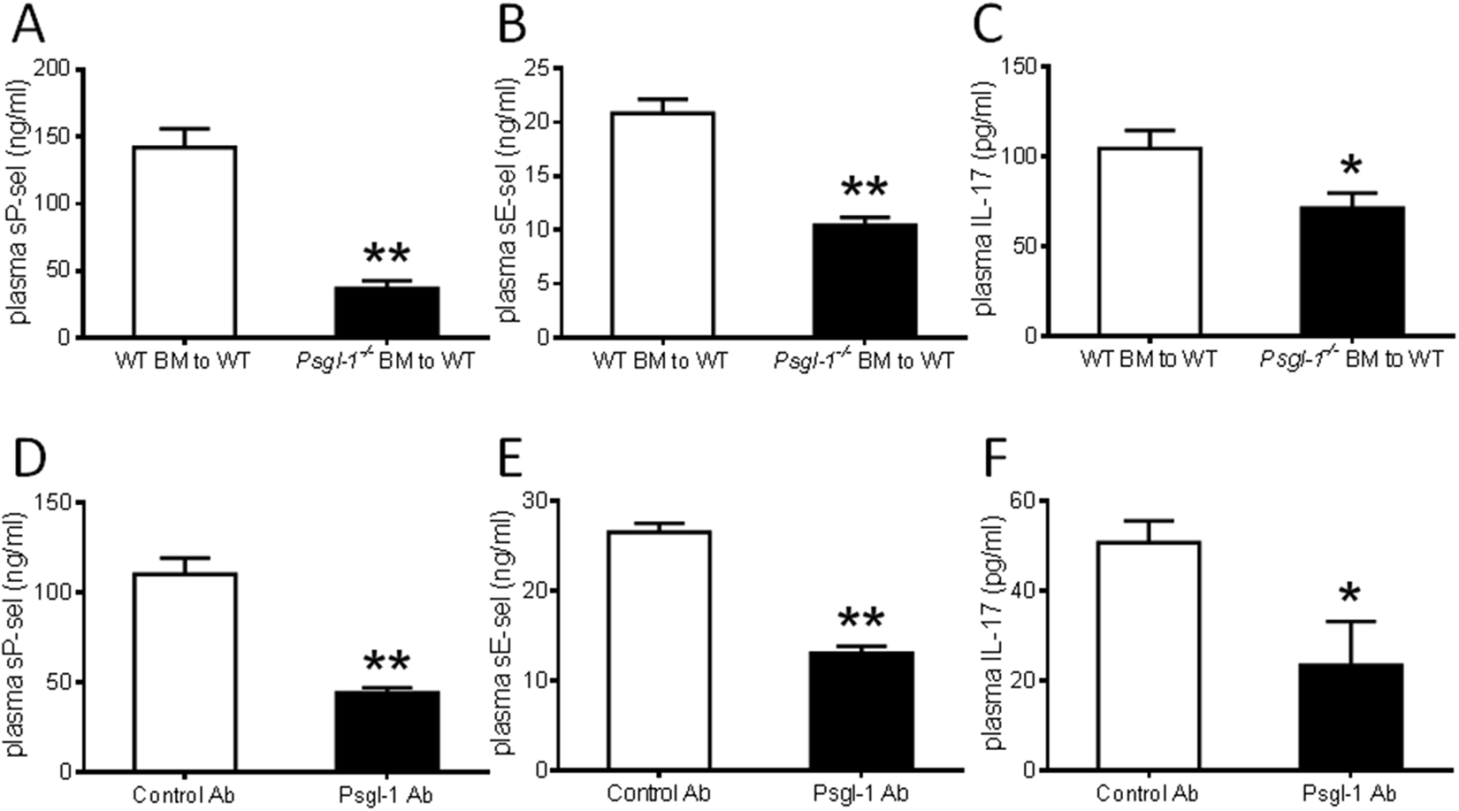
Levels of plasma factors after cumulative infusion of Ang II after bone marrow transplantation or Psgl-1 antibody treatment (n = 5 mice per group). (**A**,**B**,**C)** Levels of sP-sel (**A**), sE-sel (**B**), and IL-17 (**C**) in WT mice receiving WT bone marrow (BM) and WT mice receiving *Psgl-1*^−/−^ BM. (**D**,**E**,**F**) Levels of sP-sel (**D**), sE-sel (**E**), and IL-17(**F**) in WT mice receiving control Ab and WT mice receiving Psgl-1 Ab. *P < 0.05. **P < 0.01.

In addition to leukocytes, Psgl-1 may also be expressed on endothelial cells^20^. To determine the relevant Psgl-1 cellular pool responsible for the protective effects mediated by Psgl-1 deficiency, bone marrow transplantation (BMT) was performed from *Psgl-1*^−/−^ and WT donors to WT recipients. 8 weeks after BMT, cumulative Ang II (0.2, 2, 20, 200 μg/kg) was then administrated via the jugular vein with 5 minutes intervals. As measured by carotid artery catheterization, the pressor response to Ang II was significantly reduced in WT mice receiving *Psgl-1*^−/−^ bone marrow compared to WT mice receiving WT bone marrow (Fig. 3A). The levels of sP-sel, sE-sel, and IL-17 were also significantly lower in WT mice receiving *Psgl-1*^−/−^ bone marrow (Fig. 4A,B and C) compared to corresponding control WT mice receiving WT marrow. These findings indicate that hematopoietic Psgl-1 deficiency leads to attenuation of the pressor responses to Ang II.

### Therapeutic targeting of Psgl-1 with antibody

To directly test a therapeutic strategy of Psgl-1 blockade, an IgG control or neutralizing anti- Psgl-1 antibody was injected into WT mice intraperitoneally. 16 hours later, blood pressure response to acute Ang II infusion was evaluated as before in the common carotid artery. The pressor responses to Ang II were significantly reduced in WT mice receiving the Psgl-1 antibody compared to WT mice receiving the control antibody (Fig. 3B). The levels of sP-sel, sE-sel, and IL-17 were also significantly lower in WT mice receiving the anti-Psgl-1 antibody compared to mice receiving the control antibody (Fig. 4D,E, and F).

### IL-17 replacement restores effect of Psgl-1 on blood pressure

**Figure 5:**
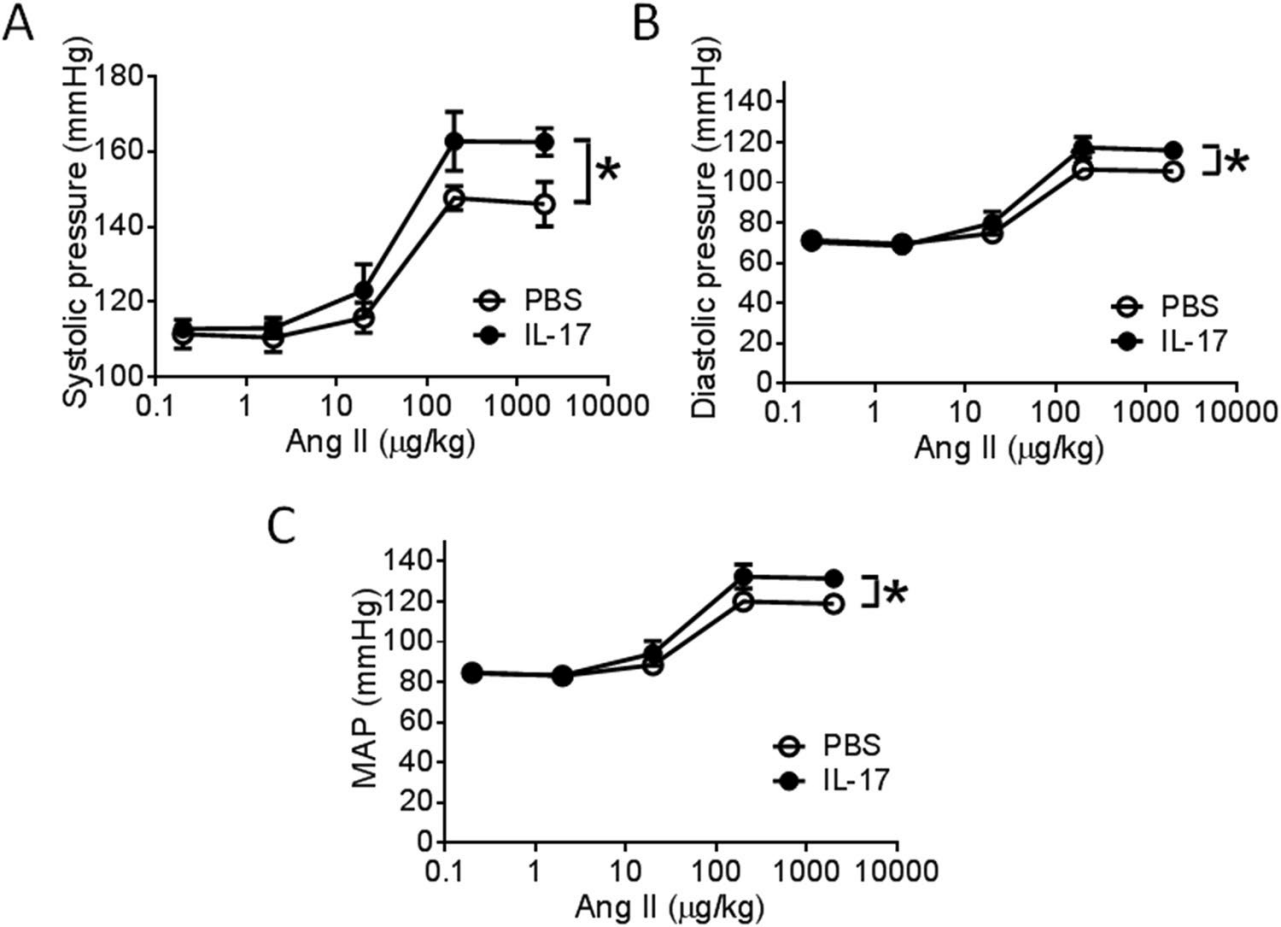
The effect of IL-17 treatment on pressor responses to Ang II in *Psgl-1*^−/−^ mice (n = 5 mice per group). Pooled data of systolic blood pressure (**A**), diastolic blood pressure (**B**), and mean arterial pressure response (**C**) to acute infusion of Ang II after PBS or IL-17 treatment in *Psgl-1*^−/−^ mice. *P < 0.05.

IL-17 deficiency states have been shown to attenuate the pressor effect of Ang II^4^. To determine if reduced IL-17 is involved in the protection conferred by Psgl-1 deficiency on Ang II-induced blood pressure elevation, IL-17 or PBS was administered to *Psgl-1*^−/−^ mice. 5 hours later, the pressor response to acute infusion of Ang II was recorded via carotid artery catheterization. Levels of the systolic, diastolic and mean arterial pressure were significantly higher in *Psgl-1*^−/−^ mice injected with IL-17 compared to PBS treatment (Fig. 5A,B and C), supporting a causal role for IL-17 towards these effects.

## Discussion

Hypertension is a common, highly complex condition that promotes risk for many other diseases of the vasculature^21^. Although the underlying cause of most cases of hypertension are unknown and likely multifactorial^22^, therapies targeting RAAS are highly effect in reducing elevated blood pressure and each of the RAAS components can promote inflammatory signaling pathways although the degree to which inflammatory pathways contribute to hypertension is unclear^23^. Circulating biomarkers are associated with elevated blood pressure^24^, however, whether they mediate or represent biomarkers of RAAS activation is unknown.

Ang II is commonly used to induce hypertension in preclinical models^25^. Biomarkers of inflammation are associated with Ang II and inflammatory pathways are activated in multiple cell types^26^. A role for leukocytes in the regulation of blood pressure following angiotensin II administration has been demonstrated. Mice deficient in various leukocyte subsets have been shown to be resistant to the pressor effects of Ang II. For example, mice deficient in T and B cells due to genetic RAG-1 deficiency are resistant to hypertension induced by angiotensin II as well as other hypertension triggers^3,27^. T cells seem to be at least partially responsible for this phenotype as adoptive reconstitution of T-cells but not B-cells restores the hypertensive effect^3^. Howerver, macrophage depletion has also been shown to blunt Ang II-induced hypertension and this effect is restored with macrophage reconstitution^28^. Taken together, these finding suggest potential cross talk between macrophages and lymphocytes in mediating the effects of angiotensin II. Multiple mediators may contribute to these effects of Ang II including the NFκB signaling pathway^11,29,30^, interleukin-6^31^, tumor necrosis factor-α^32^, macrophage colony-stimulating factor^33^ and IL-17^4^.

Since deficiency of Psgl-1 has been shown to dampen endothelial responses to cytokines via reduced activation of NFκB^10^ and to protect against obesity-induced endothelial dysfunction^13^, we therefore sought to determine whether Psgl-1 deficiency could attenuate the pressor effect of Ang II since Ang II can also activate NF**κ**B pathway via angiotensin-type 1 receptor (AT_1_R) signaling. sP-sel and sE-sel were also measured as it has been previously demonstrated that they are sensitive endothelial biomarkers of Psgl-1 activity^10^. Consistent with our hypothesis, the pressor response to angiotensin II was reduced in the setting of Psgl-1 deficiency and bone marrow transplantation experiments demonstrated this effect was mediated by the hematopoietic pool of Psgl-1. This effect was associated with reduced levels of inflammatory biomarkers supporting the link between inflammation and blood pressure elevation. While we suspect this effect is attributed to the leukocytes, we cannot rule out a contribution of platelets as Psgl-1 has been shown to be expressed by platelets^34^ and angiotensin receptor blockers have been shown to reduce platelet aggregation in hypertensive patients^35^. A neutralizing anti-Psgl-1 antibody was similarly effective in reducing the blood pressure response to Ang II highlighting a potential therapeutic application. While many mediators downstream of NFκB could contribute to these effects, IL-17 replacement was tested for its capacity to restore the hemodynamic response given previous studies implicating this cytokine as a link between AngII and inflammation-related pressor responses^4^. In support of this hypothesis, IL-17 was sufficient to restore the hemodynamic response to Ang II.

In conclusion, hematopoietic Psgl-1 regulates endothelial activation and IL-17 production in response to Ang II. These effects are associated with reduced acute and chronic pressor responses to Ang II that are restored by replacement of IL-17. Therapeutic targeting of Psgl-1 or downstream effectors such as IL-17 may be beneficial in certain subgroups of patients with hypertension.

## References

[1] Chae CU, Lee RT, Rifai N, Ridker PM (2001). Blood pressure and inflammation in apparently healthy men. Hypertension.

[2] Nakae S (2002). Antigen-specific T cell sensitization is impaired in IL-17-deficient mice, causing suppression of allergic cellular and humoral responses. Immunity.

[3] Guzik TJ (2007). Role of the T cell in the genesis of angiotensin II induced hypertension and vascular dysfunction. J Exp Med.

[4] Madhur MS (2010). Interleukin 17 promotes angiotensin II-induced hypertension and vascular dysfunction. Hypertension.

[5] Shahrara S, Pickens SR, Dorfleutner A, Pope RM (2009). IL-17 induces monocyte migration in rheumatoid arthritis. J Immunol.

[6] Zhang Z (2009). Interleukin-17 causes neutrophil mediated inflammation in ovalbumin-induced uveitis in DO11.10 mice. Cytokine.

[7] Ruddy MJ (2004). Functional cooperation between interleukin-17 and tumor necrosis factor-alpha is mediated by CCAAT/enhancer-binding protein family members. J Biol Chem.

[8] McEver RP (2001). Adhesive interactions of leukocytes, platelets, and the vessel wall during hemostasis and inflammation. Thromb Haemost.

[9] Brown JB (2012). P-selectin glycoprotein ligand-1 is needed for sequential recruitment of T-helper 1 (Th1) and local generation of Th17 T cells in dextran sodium sulfate (DSS) colitis. Inflammatory bowel diseases.

[10] Russo HM (2010). P-selectin glycoprotein ligand-1 regulates adhesive properties of the endothelium and leukocyte trafficking into adipose tissue. Circ Res.

[11] Brasier AR, Jamaluddin M, Han Y, Patterson C, Runge MS (2000). Angiotensin II induces gene transcription through cell-type-dependent effects on the nuclear factor-kappaB (NF-kappaB) transcription factor. Molecular and cellular biochemistry.

[12] Wang H (2015). Renal denervation attenuates progression of atherosclerosis in apolipoprotein E-deficient mice independent of blood pressure lowering. Hypertension.

[13] Wang H (2012). Obesity-induced endothelial dysfunction is prevented by deficiency of P-selectin glycoprotein ligand-1. Diabetes.

[14] Nguyen H (2013). Interleukin-17 causes Rho-kinase-mediated endothelial dysfunction and hypertension. Cardiovascular research.

[15] Bodary PF, Westrick RJ, Wickenheiser KJ, Shen Y, Eitzman DT (2002). Effect of leptin on arterial thrombosis following vascular injury in mice. JAMA.

[16] Pan J, McEver RP (1995). Regulation of the human P-selectin promoter by Bcl-3 and specific homodimeric members of the NF-kappa B/Rel family. J Biol Chem.

[17] Morel JC, Park CC, Woods JM, Koch AE (2001). A novel role for interleukin-18 in adhesion molecule induction through NF kappa B and phosphatidylinositol (PI) 3-kinase-dependent signal transduction pathways. J Biol Chem.

[18] Powolny-Budnicka I (2011). RelA and RelB transcription factors in distinct thymocyte populations control lymphotoxin-dependent interleukin-17 production in gammadelta T cells. Immunity.

[19] Ma HY (2016). The role of IL-17 signaling in regulation of the liver-brain axis and intestinal permeability in Alcoholic Liver Disease. Curr Pathobiol Rep.

[20] da Costa Martins P (2007). P-selectin glycoprotein ligand-1 is expressed on endothelial cells and mediates monocyte adhesion to activated endothelium. Arterioscler Thromb Vasc Biol.

[21] Forouzanfar MH (2017). Global Burden of Hypertension and Systolic Blood Pressure of at Least 110 to 115 mm Hg, 1990-2015. JAMA.

[22] Dominiczak AF (2000). Genes and hypertension: from gene mapping in experimental models to vascular gene transfer strategies. Hypertension.

[23] Luft FC (2001). Angiotensin, inflammation, hypertension, and cardiovascular disease. Current hypertension reports.

[24] Blake GJ, Rifai N, Buring JE, Ridker PM (2003). Blood pressure, C-reactive protein, and risk of future cardiovascular events. Circulation.

[25] Lohmeier TE (2012). Angiotensin II infusion model of hypertension: is there an important sympathetic component?. Hypertension.

[26] Dinh QN, Drummond GR, Sobey CG, Chrissobolis S (2014). Roles of inflammation, oxidative stress, and vascular dysfunction in hypertension. BioMed research international.

[27] Marvar PJ (2012). T lymphocytes and vascular inflammation contribute to stress-dependent hypertension. Biological psychiatry.

[28] Wenzel P (2011). Lysozyme M-positive monocytes mediate angiotensin II-induced arterial hypertension and vascular dysfunction. Circulation.

[29] Ruiz-Ortega M, Lorenzo O, Ruperez M, Suzuki Y, Egido J (2001). Angiotensin II activates nuclear transcription factor-kappaB in aorta of normal rats and in vascular smooth muscle cells of AT1 knockout mice. Nephrology, dialysis, transplantation: official publication of the European Dialysis and Transplant Association - European Renal Association.

[30] Rodriguez-Iturbe B (2005). Early and sustained inhibition of nuclear factor-kappaB prevents hypertension in spontaneously hypertensive rats. The Journal of pharmacology and experimental therapeutics.

[31] Zhang W (2012). Interleukin 6 underlies angiotensin II-induced hypertension and chronic renal damage. Hypertension.

[32] Sriramula S, Haque M, Majid DS, Francis J (2008). Involvement of tumor necrosis factor-alpha in angiotensin II-mediated effects on salt appetite, hypertension, and cardiac hypertrophy. Hypertension.

[33] De Ciuceis C (2005). Reduced vascular remodeling, endothelial dysfunction, and oxidative stress in resistance arteries of angiotensin II-infused macrophage colony-stimulating factor-deficient mice: evidence for a role in inflammation in angiotensin-induced vascular injury. Arterioscler Thromb Vasc Biol.

[34] Frenette PS (2000). P-Selectin glycoprotein ligand 1 (PSGL-1) is expressed on platelets and can mediate platelet-endothelial interactions *in vivo*. J Exp Med.

[35] Suresh A (2016). A Pilot Study on the Effect of Angiotensin Receptor Blockers on Platelet Aggregation in Hypertensive Patients- A Prospective Observational Study. Journal of clinical and diagnostic research: JCDR.

